# Applications of LiDAR in Agriculture and Future Research Directions

**DOI:** 10.3390/jimaging9030057

**Published:** 2023-02-24

**Authors:** Sourabhi Debnath, Manoranjan Paul, Tanmoy Debnath

**Affiliations:** School of Computing, Mathematics and Engineering, Charles Sturt University, Bathurst, NSW 2795, Australia

**Keywords:** LiDAR, agriculture, canopy volume, biomass, phenotype

## Abstract

Light detection and ranging (LiDAR) sensors have accrued an ever-increasing presence in the agricultural sector due to their non-destructive mode of capturing data. LiDAR sensors emit pulsed light waves that return to the sensor upon bouncing off surrounding objects. The distances that the pulses travel are calculated by measuring the time for all pulses to return to the source. There are many reported applications of the data obtained from LiDAR in agricultural sectors. LiDAR sensors are widely used to measure agricultural landscaping and topography and the structural characteristics of trees such as leaf area index and canopy volume; they are also used for crop biomass estimation, phenotype characterisation, crop growth, etc. A LiDAR-based system and LiDAR data can also be used to measure spray drift and detect soil properties. It has also been proposed in the literature that crop damage detection and yield prediction can also be obtained with LiDAR data. This review focuses on different LiDAR-based system applications and data obtained from LiDAR in agricultural sectors. Comparisons of aspects of LiDAR data in different agricultural applications are also provided. Furthermore, future research directions based on this emerging technology are also presented in this review.

## 1. Introduction

Light detection and ranging (LiDAR) sensors have been considered one of the most important technologies for agricultural sectors because they are a form of non-destructive remote sensing technology that is not affected by light conditions. They also provide information with higher accuracy compared to other digital sensing technologies. LiDAR is based on remote sensing and can calculate the distance between the sensor and the target by emitting an electromagnetic signal as a laser beam towards a given direction. The elapsed time between emitting and receiving the laser beam is employed to calculate the target’s distance. LiDAR data sets are captured using special sensors from the air or the ground, resulting in a set of point clouds all having x, y and z positions relative to a coordinate [1]. A tree’s geometrical and structural parameters, such as canopy areas and volumes, leaf area index, etc., can also be obtained by direct, semidirect and indirect methods. In direct methods, the features are obtained by measuring heights and widths, counting, and destructive sampling, such as plucking leaves from plants [2]. Hence, the data collection process is time-consuming, labour-intensive, costly and can lead to human error. In a semidirect method such as Wilson’s inclined point quadrat method [2], the number of leaf contacts is counted using probes in the vegetation canopy. Indirect methods use passive optical devices that are based on a gap fraction method. However, these methods have limitations in spatial explicitness and accuracy.

There are also digital sensing technologies such as light spectrum analysis, image analysis techniques, infrared thermography, stereoscopy photography, and optical ranging to measure and detect plants’ structural characterisation. These techniques are affected by light conditions [3]. There are topological, bathymetric and terrestrial LiDARs. The basic working principle of these LiDARs is the same. Topological LiDAR generally uses a near-infrared laser to measure the distance of objects on the ground. A bathymetric LiDAR uses a green wavelength of 532 nm that can penetrate water to map seafloor and riverbed elevations [4]. On the other hand, a terrestrial LiDAR is usually mounted on a moving vehicle or a tripod to collect data of surrounding surfaces and objects.

There are potential applications of LiDAR in sectors such as agricultural, flood modelling, coastline management, archaeology, oceanography, navigation and collision avoidance in autonomous vehicles, physics and astronomy, military and law enforcement, etc. [5]. Researchers found that LiDAR-based technology can also be used to detect CO_2_ in Earth’s atmosphere [6,7,8], allowing researchers to develop pollution modelling to monitor and reduce imperfect builds effectively. LiDAR data have also been used to create fuel maps to study bushfires [9]. Although there are multidisciplinary applications of LiDAR, the primary goal of this paper is to provide a comprehensive review of the applications of LiDAR in the agricultural sector. LiDAR-based systems gained attention from researchers for non-destructive remote sensing applications in agricultural sectors that are not affected by light conditions [3]. Researchers are focusing on developing technologies based on LiDAR sensors. LiDAR is widely used in agricultural landscaping to map water flow and water flow direction and the trees in an orchard. It was reported that using LiDAR data, it is possible to visualise, measure and map out variations such as slope, aspect, soil erosion and elevation of agricultural lands. The information accrued from LiDAR data can be used for planning to manage agricultural lands effectively [10]. The measurement of canopy volume [11], leaf area index [12], tree area index [13], biomass [14], etc., also assist in estimating fertiliser and pesticide dose [11,15], biomass accumulation and carbon storage, etc. Understanding the structural characteristics of canopy phenological stages and phenotype characterisation [16,17] are also crucial. These influence photosynthesis, growth, plant development, health, yield potential, CO_2_ sequestration and evapotranspiration. Hence, this understanding plays a crucial role in maintaining terrestrial and aboveground agronomic systems [18,19]. With such information, the different characteristics and appropriate management for each zone of agricultural zones can be determined.

The working principle of LiDAR is described in Section 2, information regarding different types of LiDAR is presented in Section 2.1, different applications of LiDAR data are reviewed in Section 3, data processing is elaborated in Section 4 and future directions of research in the application of LiDAR data are discussed in Section 5.

The key contributions of this paper are as follows:

- The applications of LiDAR data in agriculture of the last 14 years (2008 to 2022) are discussed. We believe that this can help readers, especially newcomers to this area, understand the trend of the application of LiDAR in the agricultural sector;
- Comparisons of aspects of LiDAR data in different agricultural applications along with various data processing aspects are also provided;
- A discussion on future research directions of LiDAR-based system are also presented.

## 2. How LiDAR Works

**Figure 1:**
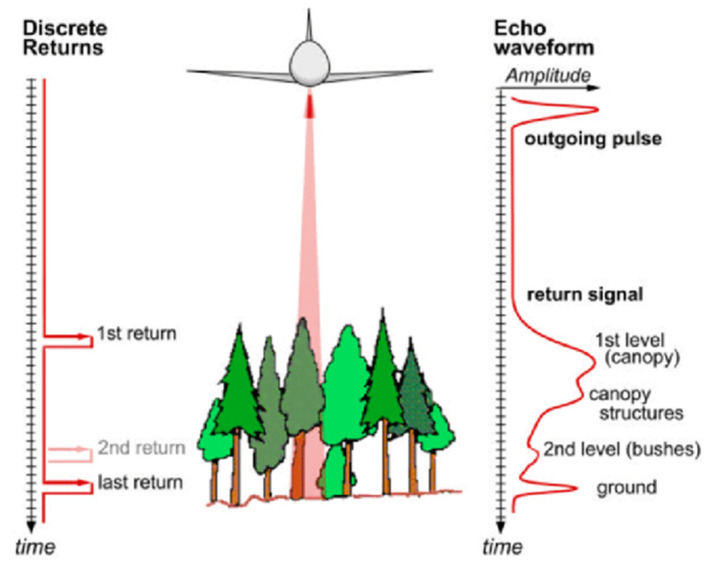
Illustration of laser beam outgoing and return signals (discrete and full-waveform) of LiDAR. Reprinted with permission from Ref. [9]. Copyright 2009 Ferraz, A.

The working principle of a LiDAR-based system is presented in Figure 1. Figure 1 shows that when photons of light energy from LiDAR (e.g., airborne LiDAR) hit various objects (e.g., roads, buildings, tree branches, bridges, etc.), some light reflects off those objects and returns to the sensor. Depending on the size of the objects and surrounding gaps, some light continues down towards the ground. This phenomenon results in multiple reflections, and a waveform is created by the distribution of energy that returns to the sensor, which is then recorded. The distribution of energy peaks is created within the regions where the amount of light energy returned to the sensor is high [20].

Based on how the returns are recorded, there are discrete return LiDAR and full-waveform LiDAR. Both a discrete return LiDAR system and a full-waveform LiDAR system can record returns. Discrete points for the peaks in the waveform curve are recorded in a discrete return LiDAR system. On the other hand, the returned light energy distribution is recorded in a complete waveform LiDAR system that might obtain the distance between the LiDAR sensor and the objects. This distance can be measured by time-of-flight (TOF) or phase shift measurement (PMS) methods. Time-of-flight (ToF) calculates the distance between a LiDAR sensor and objects of interest that reflect the original signal emanating from the sensor by recording the time difference between the original and reflected signals. A LiDAR system utilises light in the shape of a pulsed laser in TOF. The start time of the emitted pulse and the time of the reflected pulse hitting the sensor are recorded to calculate the time it takes for the pulse to return to the LiDAR source [21,22,23]. Thus, Equation (1) can be used to measure how far an individual photon has travelled to and from an object.

d = c t/2,(1)

where d = distance to the object, c = light speed and t = time between the emitted and detected light.

Light power is modulated at a constant frequency in PMS, where a continuous light source is employed. Therefore, the modulated light can be characterized as sinusoidal (time and laser power on the x and y axes, respectively) in nature. The difference in radians of the waves’ peaks and the object’s distance can be found by Equation (2) [23].

d = cΔΦ/2πf (2)

where d = distance, c = light speed, ΔΦ = phase difference and f = frequency of the modulated power.

The distance obtained from TOF or PMS is then converted to elevation. Elevation information is then used to represent ground objects.

### 2.1. Types of LiDAR

There are mainly airborne and terrestrial LiDAR systems, and they are split according to functionality. Airborne LiDAR can be installed on a drone or helicopter and is classified as topological LiDAR or bathymetric LiDAR. Topological LiDAR utilises a near-infrared laser for land mapping and bathymetric LiDAR employs water-penetrating green light for seafloor or riverbed elevation measurements [21,24].

Most airborne LiDAR systems consist of LiDAR sensors, data storage devices, an on-board computer, a global positioning system (GPS) and an inertial measurement unit (IMU) [25]. The GPS is responsible for determining the aircraft’s location and the IMU records the precise orientation of the sensor. Terrestrial LiDAR systems are generally mounted on vehicles or a tripod on the ground to obtain the required information. Terrestrial LiDAR uses mirrors to capture data from several directions [26]. Terrestrial LiDAR can also be used to obtain data for areas such as under tree canopies, where airborne LiDAR cannot reach. Like airborne LiDAR, various objects reflect the pulsed laser, and the device is used to calculate their distance from the object.

**Figure 2:**
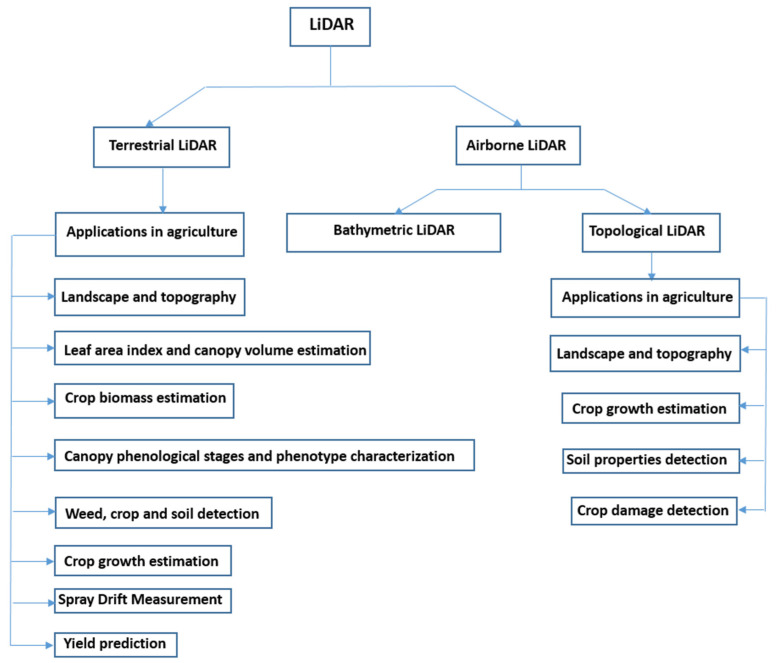
A chart of applications of LiDAR in the agricultural sector.

Figure 2 presents a chart of different applications of LiDAR in agriculture. LiDAR systems can be broadly classified into terrestrial and airborne categories. In the agricultural sector, terrestrial LiDAR is primarily used for determining landscape and topography, leaf area index and canopy volume estimation; crop biomass estimation; canopy phenological stage and phenotype characterization; weed, crop and soil detection; crop growth estimation; spray drift measurement; yield prediction; etc. Airborne LiDAR can be classified as bathymetric LiDAR and topological LiDAR. There are not yet many reports of usage of bathymetric LiDAR in agriculture. Topological LiDAR is primarily used for determining landscape and topography, crop growth estimation, soil property detection, crop damage detection, etc.

## 3. Applications of LiDAR

LiDAR generally records GPS time; scan angle and direction; x, y and z position information; intensity; return number; the number of returns; and point classification values [27]. These stored data sets are then used to obtain the target objects’ height, volume and area information. The obtained information is then used for determining the landscape and different physical characteristics of the trees, crop biomass estimation, spray drift measurement, soil property detection, yield prediction, crop damage detection, etc.

In the following sections, different applications of LiDAR data sets in agricultural settings are discussed.

### 3.1. Landscape and Topography

LiDAR sensors have been widely used to determine the landscape and topography of agricultural land for planning and managing agriculture. Applications of LiDAR data in landscaping and topography determination are presented below.

#### 3.1.1. Ditch Network Detection

J.S. Bailly et al. used airborne LiDAR to collect Mediterranean vineyard data to detect a ditch network [28]. In the study, the proposed approaches were based on the hypothesis that ditches were located at field boundaries. Therefore, a concavity within an elevation profile would be directed across the ditch. In the proposed method, elevation profiles were estimated on a set of pre-located sites from raw data. The derivation of profile for concavity indicators was also applied with the aid of 1D wavelet transforms (DWT) and a watershed algorithm. In addition, the classification and regression tree (CART) segmentation method was used to differentiate ditches from non-ditches. It was reported that the overall accuracies of DWT and the watershed algorithm were 71.3% and 71.7%, respectively, with an around 50% mean ditch omission rate and approximately 15% mean ditch commission rate for both DWT and watershed. The results show that ditch omission rates were higher for high-vegetation sites, but detection rates were around 75% when little vegetation existed.

#### 3.1.2. Terrace Group Detection

Mark D. McCoy et al. proposed slope contrast mapping to identify agricultural terrace groups from a LiDAR data set [29]. Their study used a geographic information system (GIS) model to recognise flat-to-low slope areas between drainages and compare results with slope contrast mapping. A slope contrast model (SCM) was used to evaluate top-of-canopy and ground digital elevation models (DEMs) from the LiDAR point cloud data. The SCM considered a vertical back slope of 900 to be created by a cut out of a natural slope. Side slopes of 900 were created by cutting and filling. The downslope was composed of fill held in place by a retaining face [29]. After obtaining the digital elevation model (DEM), the DEM was transformed into a slope raster. The slope raster then was turned into a classified raster by dividing cells into flat, low and high slope categories. It was reported that though the proposed SCM could map naturally flat areas estimating where irrigated agriculture expanded in the past, it was less effective than the GIS model.

#### 3.1.3. Erosion Detection

A hydro-geomorphological analysis to identify terraces and road-induced erosion of vineyards was proposed by Tarolli et al. [10]. At first, the relative path impact index (RPII) was derived from the digital terrain model (DTM) obtained from airborne and terrestrial LiDAR scanners to identify terraces and road-induced erosions. The index’s statistical threshold was then used to mark the critical areas for surface erosion in the terraced vineyard.

**Figure 3:**
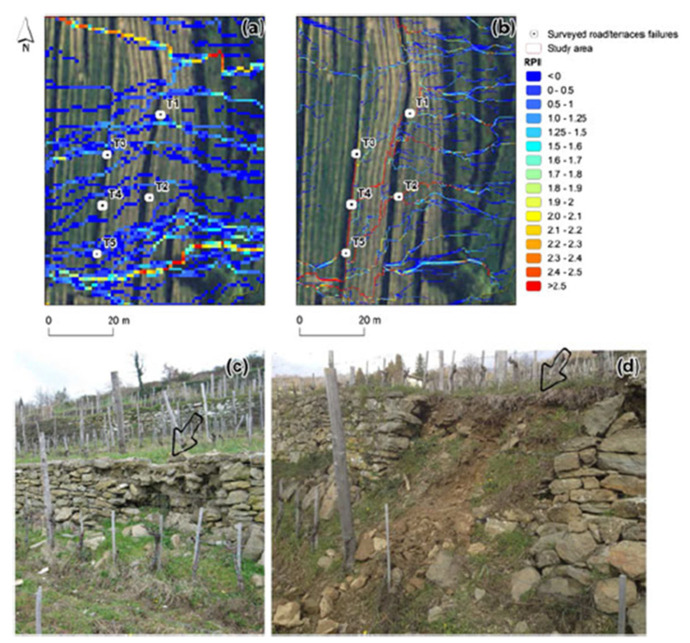
RPII maps derived from the 1 m ALS DTM (**a**) and the 0·2 m TLS (**b**), highlighting the flow modifications T1 (**c**) and T5 (**d**). Reprinted with permission from Ref. [10]. Copyright 2015 Wiley.

Different soil conservation measures were simulated using the index and the defined thresholds to determine the optimal solution to reduce collapsing or erosion in vineyards induced by agricultural roads and terraces. Figure 3 shows that though using RPII, the road size was captured correctly using a 1 m airborne LiDAR scanner (ALS) DTM, but the terrace failures T1 to T2 could not be accurately identified to characterise the flow alternations correctly. On the other hand, for a 0·2 m terrestrial LiDAR scanner (TLS) DTM, the RPII produced more accurate results and depicted all the surveyed failures (T1 to T5) correctly.

#### 3.1.4. Overland Flow Detection

Jake Galzki et al. proposed that by applying LiDAR-based terrain attributes, fine-scale areas consisting of overland flow could be identified [30]. Their study areas were hydrologically connected to surface water either by an overland flow path or subsurface drainage along agricultural ditches. These focused areas had a higher possibility of contributing to surface water quality degradation by conveying contaminants, nutrients and pesticides to nearby water sources. Precision conservation techniques were used in this proposed method to obtain LiDAR data with acceptable resolution. LiDAR data were converted into hydrologically corrected DEMs with 1 m grid cell resolution and then resampled to a 3 m DEM. Various terrain attributes, e.g., slope, flow accumulation and stream power index, were then derived from the DEMs. It was reported that out of the 32 most prominent gullies, 3 m LiDAR correctly identified 31, whereas 30 m LiDAR could identify only 7 [30].

#### 3.1.5. Parcel Detection

In another application in agricultural landscaping, Adam J. Mathews et al. proposed a method to extract and classify vineyard land-use parcels and parcel boundaries. The study was carried out in three different vineyard sites. A DTM, digital surface model (DSM) and normalised digital surface model (nDSM) were obtained from the raw LiDAR data set. In this proposed method, the nDSM was developed using airborne LiDAR data of inter-row spaces, canopy features and a focal statistics method [31]. The study presented that though the mean accuracies of correctly classified vineyard area and parcel delineations were 97.55% and 88.79%, respectively, this method had limitations when it came to distinguishing separated but adjacent vine parcels in the vicinity. In the proposed method, a 12 × 12 analysis window was used. The scan window size was a function of the geometry of vine rows, spacing and the DSM’s resolution.

#### 3.1.6. Canopy Openness Detection

In a different type of application of LiDAR data, Collin et al. proposed a method where airborne LiDAR data were employed to estimate canopy openness to model solar radiation in terms of light penetration index (LPI) [32]. This index determines the probability that a direct beam of light will pass through the plants and touch the earth. First, the LiDAR point clouds stipulated the probability of light reaching the ground; then, this probability was determined by considering the LPI. Thus, the laser was considered a substitute for sun rays embracing the ground. In this proposed model, the GRASS GIS r.sun solar model was used to develop the subcanopy solar radiation model (SSR) from the LiDAR dataset. The GRASS GIS r.sun was a clear sky solar model that considered topographic angles, shading, DEM, Julian day, time-step and Linke turbidity index. The SSR model utilised both direct and indirect field measurements to estimate subcanopy radiation.

Pyranometers were employed for direct measurements of global and diffuse radiation, whereas hemispherical photographs and a gap light analyzer (GLA) were used for indirect measurements. The SSR could predict direct radiation better than diffuse radiation. A simple linear regression (SLR) analysis found that SSR and GLA for total solar radiation predictions matched field measurements with R^2^ = 0.92 and R^2^ = 0.692, respectively. However, predictions were not highly accurate in diffuse radiation for both SSR and GLA. Canopy openness obtained from GLA and LPI were correlated with R^2^ = 0.768. Table 1 presents the comparisons of various aspects of LiDAR data in determining agricultural landscape and topography.

### 3.2. Leaf Area Index and Canopy Volume

Application of LiDAR was also reported for measuring leaf area index (LAI) and canopy volume. A tractor-mounted 2D LiDAR was proposed by Rosell et al. to make recordings of 3D tree row structures in pear and apple orchards and grape vineyards [19]. In this method, the 3D point cloud was visualised with a computer-aided design [18]. Scanner data were used to calculate the volumes and leaf area of trees to probe the suitability of laser sensors to characterise vegetation. Foliage areas and plant volume were also compared with leaf areas employing SLR analysis [19]. Data were collected using a LiDAR sensor regarding the different growth stages of crops and before and after the defoliation of designated trees on both sides of the crop rows. Two methods were solicited to determine leaf area. In the first method, the relationship between plant volume measured by LiDAR and its corresponding manually measured total foliar area was considered. The second method formulated by Walklate et al. used Beer’s law and the tree area index (TAI) [13]. Here, the TAI was defined as the ratio between crop detected area and ground area. The SLR analysis found the coefficient of determination (R^2^) between plant volume and LAI for pear orchards, apple orchards and vineyards to be R^2^ = 0.8422, R^2^ = 0.814 and R^2^ = 0.8058, respectively. However, for vineyards, the coefficient of determination between LAI and TAI was reported to be R^2^ = 0.9194 [19].

On the other hand, by comparing the results obtained from LiDAR sensors with ultrasonic and traditional manual canopy measurement procedures, Jordi Llorens et al. found that crop width and volume calculated from LiDAR sensor data presented lower values of R^2^ than those obtained from ultrasonic and manual processes [11]. The correlations between LAI and canopy volume using ultrasonic and LiDAR sensors were found with R^2^ = 0.51 and 0.21, respectively. However, the correlation between calculated volumes with ultrasonic and LiDAR was obtained with R^2^ = 0.56. It was reported that LiDAR sensors possessed a better ability to detect gaps in the canopy but required software for LiDAR data analysis to obtain accurate information.

Furthermore, in an experimental study of J. Arno’ et al., LAI was estimated from LiDAR data [33]. The measured LAIs with low, medium and high vigour zones were obtained using cluster analysis. LAI was determined considering the projected left, right and top surface and the ratio between leaf area and projected envelop area. Here, LiDAR overestimated LAI compared to measuring LAI in all the three vigour zones, with decreasing differences with more leaf surfaces. It was reported that if the maximum distance between scans along the rows did not exceed 15 m and the scanned length was 1 m, the LiDAR system could be used intermittently.

J. Arno et al. also calculated LAI considering TAI using LiDAR sensor data in another study. The TAI was formulated using a Poisson distribution. Besides TAI, tree height, cross-sectional area and canopy volume were also calculated from LiDAR sensor data [12]. The relationship between measured LAI and the parameters mentioned above was obtained with SLR analysis. SLR analysis estimated LAI from canopy tree height, cross-sectional area, and volume for 1 m long sections of the total row width with R^2^ = 0.62, 0.72 and 0.81, respectively. Furthermore, the LAI estimation obtained from the TAI was with R^2^ = 0.92 for the same length. In this proposed method, the non-random distribution of leaves and the inability of LiDAR to distinguish leaf and woody materials affected the accuracy of LAI estimation.

On the other hand, using a remote-controlled prototype robot consisting of a LiDAR scanner, C. Poblete-Echeverría et al. obtained data to estimate LAI to study vineyard growth regarding seasonal progression and environmental progression [34]. Mesh surface area (MSA) was also evaluated from LiDAR data to demonstrate the usefulness of LiDAR in the estimation of LAI. SLR analyses were applied to obtain the correlation between actual LAI and the MSA values. The results demonstrate a correlation between MSA and LAI, with an R^2^ of 0.798 and an RMSE of 0.05 m. Table 2 presents the comparisons of various aspects of LiDAR data in measuring leaf area index and canopy volume.

### 3.3. Crop Biomass Estimation

The application of LiDAR sensors to estimate crop biomass was also reported. LiDAR-based volumetric modelling was also proposed by Keightley et al. to measure the volume of the biomass of grapevine [35]. LiDAR data were collected from grapevine (mounted on a turntable) trunks and cordons and used to generate 3D models of that vine’s perennial woody tissue. An analogue measurement was also carried out where the vine trunks and cordons were submerged in water and then the displaced water was captured. Water weight was converted to volume after recording. The vines were dried using an oven and weighed. Volume, density and mass data were collected for each vine. An SLR analysis was also accomplished to obtain the relationship between volumes obtained from LiDAR data and analogue measurements. It was reported that the standard error dropped with the increasing number of added scans to the volume calculation. Volumes calculated from the LiDAR data ranged between 1.31 and 10.61 L, whereas volumes obtained from the analogue measurement ranged between 0.83 and 5.05 L. The SLR of analogue volume on LiDAR-based volume found that slope values ranged between 0.43 and 0.54. The Pearson product-moment correlation coefficient (PMCC) had a range from 0.73 to 0.97. LiDAR-based volume estimates were more significant than those calculated using the analogue method presented by the y-intercepts. The regression slope values with the RMSE ranged from 4.23 to 1.45. These results suggest that the volume calculated from LiDAR data for smaller vines having smaller trunk and cordon diameters had a more significant deviation than for larger vines compared to volumes obtained from analogue measurement. It was noted that the deviation in volume decreased when vine size increased. The LiDAR system used in the study had instrumental uncertainty of ±4 mm and measured features with a 10 mm diameter [35]. Hence, measuring smaller vines required analysis closer to the accuracy limit. Furthermore, complex plant geometry was also one of the factors that affected the accuracy of the results.

**Figure 4:**
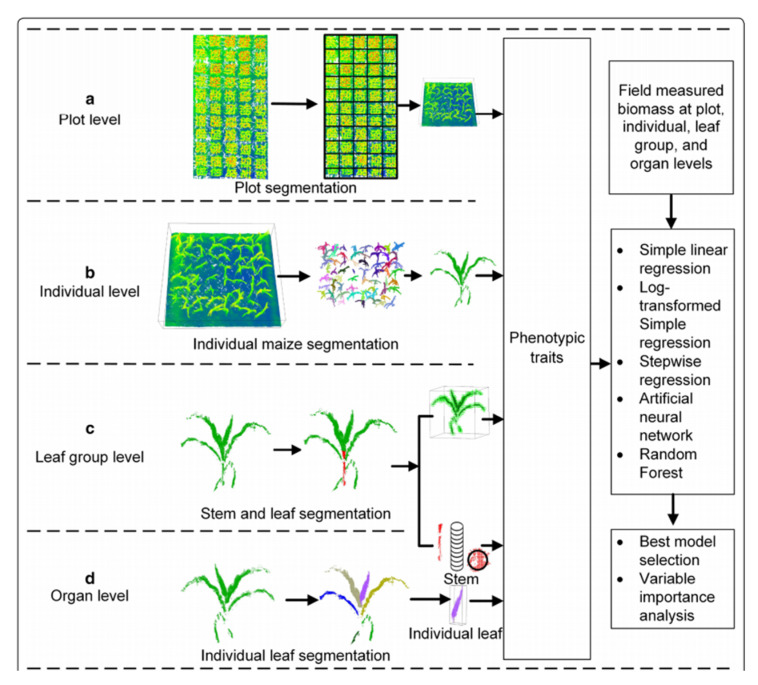
Workflow of the biomass estimation and best model and phenotypic trait selection at (**a**) plot level, (**b**) individual segmentation, (**c**) stem–leaf segmentation and (**d**) individual leaf segmentation. Reprint from Ref. [14].

On the other hand, Shichao Jin et al. estimated field maize biomass at the plot, i.e., plot segmentation; individual plant, i.e., individual maize segmentation; leaf group, i.e., stem and leaf segmentation; and individual organ, i.e., individual leaf or stem, levels, as shown in Figure 4 [14]. LiDAR data were collected at four different levels. The data were used to extract different phenotypic traits such as 1D traits (e.g., height), 2D traits (e.g., canopy cover) and 3D traits (e.g., volume). All phenotypic traits were used to build SLR, log-transformed simple regression (LSR), stepwise multiple regression (SMR), an artificial neural network (ANN) and random forest regression (RFR) to determine the suitable methods and phenotypic traits for biomass estimation. The estimated biomass for all the four levels calculated from the LiDAR data were compared with the field-measured data of corresponding levels. It was reported that at the plot level, the best LSR model and SLR model were both built with variable H84 (i.e., the 80% quantile height), which had R^2^ of 0.79 and 0.80, respectively. The R^2^ and RMSE of SMR, ANN and RFR were 0.80 and 179.77 g, 0.68 and 222.40 g and 0.79 and 150.14 g, respectively. Again, at the individual plant level, the best LSR and SLR models had R^2^ of 0.93 and 0.96, respectively.

Meanwhile, the R^2^ and RMSE of SMR, ANN and RFR were also high and at rates of 0.94 and 10.29 g, 0.93 and 10.75 g, and 0.94 and 10.19 g, respectively. The best LSR and SLR models were built with height and 3DPI variables in the leaf group level and had R^2^ of 0.95 and 0.92, respectively. The R^2^ and RMSE of SMR, ANN and RFR were 0.97 and 2.41 g, 0.97 and 2.22 g, and 0.97 and 2.34 g, respectively. Furthermore, the best LSR and SLR models were built at the stem level with stem height variables and R^2^ of 0.93 and 0.94. The R^2^ and RMSE of SMR, ANN and RFR were also high and were 0.94 and 6.40 g, 0.95 and 5.68 g, and 0.95 and 5.81 g, respectively. On the other hand, at the individual leaf level, the best LSR and SLR models had leaf area variables with R^2^ of 0.84 and 0.84, respectively. The R^2^ and RMSE of SMR, ANN and RFR were 0.86 and 0.62 g, 0.86 and 0.67 g, and 0.78 and 0.84 g, respectively. From the data mentioned above, it was found that the value of R^2^ was greater than 0.80 at all levels for estimating maize biomass. Biomass estimation at the leaf group level was R^2^ = 0.97 with RMSE = 2.22 g in four levels [14].

Moreover, for biomass and crop nitrogen (N) estimation, Jan U.H. Eitel et al. used TLS employing a pulsed green (532 nm) laser. A green laser was utilised as its return intensity might help sense chlorophyll related attributes (e.g., crop N status). The study considered that a plant’s chlorophyll concentration affects absorbed green light. [36]. Increased green laser light is absorbed with increasing plant chlorophyll concentration, decreasing the laser return’s intensity. In this study, the green laser light reflected by the sensor was recorded by the TLS instrument. Above-ground crop mass was also estimated from TLS data. Using DSM and DTM, laser-derived vegetation volume was calculated. With the aid of computer programming, nitrogen concentration was estimated from LiDAR data. The laser-derived vegetation volume and the normalised intensity of the reflected green light were then used to calculate the nitrogen nutrition index (NNI). It was found that the accuracy of laser-derived vegetation volume decreased with increasing canopy height and volume since the possibility of the laser beam penetrating the canopy completely decreased with increasing canopy cover. From SLR analysis, relationships between observed physical proxies (e.g., crop height or volume) and TLS-derived vegetation volume for all seasons and growth stages were with R^2^ >= 0.72 and RMSE ≤ 0.68 t ha^−^^1^. The range of relationships between the actual nitrogen concentration and green laser return intensity had R^2^ = 0.10–0.75 and RMSE = 0.31–0.63% [36]. Furthermore, James D. C. Walter used LiDAR data to estimate aboveground biomass (AGB) along with canopy height (CH) of the wheat crop [37]. In the study, LiDAR-derived measurements were contrasted against AGB and CH manual measurements to assess application suitability within a breeding program. It was reported that the correlations between AGB and LiDAR projected volume (LPV) were up to Pearson’s correlation coefficient (r) of 0.86 and correlations between CH and LiDAR canopy height (LCH) were up to r = 0.94. Table 3 presents the comparison of several aspects of LiDAR data in crop biomass estimation.

### 3.4. Canopy Phenological Stages and Phenotype Characterisation

LiDAR data also were applied to study the phonological stages and phenotype characterisation of crops. Rinaldi M. et al. [16] implemented a mathematical protocol to characterise the phenological stages, i.e., BBCH stages of grapevine canopy. In the study, tree row volume (TRV), leaf wall area (LWA) and LAI were calculated. The BBCH stages were determined utilising manually collected data sets and with LiDAR scanning at each canopy phenological stage. The estimated values of TRV, LWA and LAI derived from LiDAR data were compared with manual measurements. The correlations between manual and LiDAR scan measurements of height and width were R^2^ = 0.98 and R^2^ = 0.81, respectively. The plant was measured from each side and the superposition of scans resulted in errors. Hence, the measurement of the width was less accurate than the height. Furthermore, the study demonstrated that the coefficients of determination between the estimated values of TRV or LWA and the growth stage of the vine were R^2^ = 0.99 and R^2^ = 0.95, respectively. It was also possible to monitor time-series phenotype dynamics of maize under drought stress [17]. Several parameters, e.g., plant height, projected leaf area (PLA) and plant area index (PAI) could be obtained from the terrestrial LiDAR point clouds at the individual plant level. Rinaldi M. et al. also considered voxel size when estimating PAI from LiDAR data because the voxel-based method underestimated the PAI if the voxel size was too small.

On the contrary, the method overestimated the PAI if the voxel size was large. In the study, a voxel size of 1.5 times the average point distance was effective for obtaining relatively high accuracy to estimate the PAI. The results show that the estimation accuracies of plant height, PLA and PAI obtained from LiDAR data had R^2^ of 0.96 and RMSE of 0.15 m^2^/m^2^, R^2^ of 0.92 and RMSE of 0.05 m^2^/m^2^ and R^2^ of 0.70 and RMSE of 0.15 m^2^/m^2^, respectively, compared to manual measurements [17]. During the growth period, plant height, PAI and PLA demonstrated a trend of first increasing and later decreasing. Distance-based clustering analysis was also employed. Nine, five and three of the seventeen maize varieties were grouped as low drought tolerance, medium drought tolerance and high drought tolerance, respectively.

Furthermore, to develop a high throughput phenotyping system and analyse the growth of cotton plants, Shangpeng Sun et al. [38] used terrestrial 2D LiDAR. They reconstructed a 3D model of the scanned crops. The 3D model was then used to calculate morphological characteristics such as canopy height, projected canopy area (PCA) and plant volume. In this study, experiments were performed on four cotton cultivars. First, an analysis of variance (ANOVA) test was performed to understand the effect of cultivar on height trait. Second, 3D plant reconstructions were performed for detecting the growth patterns of cotton plants of the different cultivars over time. The validation experiments indicated a correlation between LiDAR measurements and manual measurements for maximum canopy height, PCA and plant volume, with R^2^ and RMSE values of 0.97 and 0.03 m, 0.97 and 0.007 m^2^ and 0.98 and 0.011 m^3^, respectively. The highest R^2^ values between PCA and final yield for cultivars 1, 2, 3 and 4, between 88 and 109 days after planting (DAP), were 0.65, 0.83, 0.87 and 0.88, respectively. On the other hand, the maximum R^2^ values between plant volume and final yield were 0.77, 0.85, 0.84 and 0.83 on 95, 67, 74 and 74 DAP for cultivars 1, 2, 3 and 4, respectively [38]. This study suggests that PCA and plant volume could be utilised as high-throughput phenotyping tools to detect productivity differences other than canopy height.

Moreover, for high-throughput estimation in maize and sorghum crops, Suresh Thapa et al. developed a LiDAR scanner with a precision rotation stage to produce 3D point clouds of plants with a 360-degree view [39]. The obtained data were then processed for noise removal, voxelisation, triangulation and plant leaf surface reconstruction. Plant morphological characteristics such as individual and total leaf area, leaf inclination angle and angular leaf distribution were obtained after reconstructing the digital leaf surfaces. The angle measured between the plant’s leaves and its stem was considered the leaf inclination angle in the reference method. All leaves on the plant were cut after imaging and leaf area was obtained using a leaf area meter. Finally, locally weighted scatterplot smoothing (LOWESS) was applied to reconstruct the leaf surface. The R^2^ between model-derived leaf area and the reference measurement was 0.92 for maize and the mean absolute error (MAE) was 43.2 cm^2^.

On the other hand, for sorghum, the R^2^ between the two measurement sets was 0.94 and the MAE was 16.0 cm^2^. These results show that the correlation between leaf area measured using the LiDAR-based instrument and the reference methods was R^2^ > 0.91 for individual leaf area and R^2^ > 0.95 for each plant’s total leaf area. The 3D models underestimated individual leaf area in contrast to the area meter measurement because of maize’s wrinkle structures at the edges of leaves. The LiDAR-based system did not detect those. On the contrary, as sorghum leaves are smoother than maize, this introduced a lower amount of systematic error in leaf area modelling. The correlation between leaf inclination angles measured from 2D images and acquired from the 3D model for maize plants had R^2^ = 0.904 [39]. Meanwhile, for sorghum, the coefficient of determination was R^2^ = 0.723. Some limitations of this proposed method were reported, which are to be noted. In this proposed method, the maize and sorghum examined had a simple plant structure and large leaves. Hence, the structure of the leaves and plants could influence the experimental results. With this method, it could be challenging to obtain the canopy’s complete point cloud with a denser leaf structure due to occlusion. On the other hand, smaller leaves could reduce the efficiency of surface reconstruction, and if the plant’s stem is not upright, it might be difficult to remove the stem from the raw point clouds [39]. The results could also vary as parameters such as LiDAR height for point cloud generation, start and stop angles, the threshold for point cloud noise removal and the leaf number for k-means clustering in point cloud processing were inserted manually. This might not be desirable in a practical setting. Table 4 presents a comparison of various aspects of LiDAR data in canopy phenological stages and phenotype characterisation.

### 3.5. Weed, Crop and Soil Detection and Crop Growth Estimation

LiDAR data can also be used to detect weeds, crops and soil. It can also be applied to estimate crop growth and fresh aboveground weight.

#### 3.5.1. Weed, Crop and Soil Discrimination

Dionisio And’ujar et al. [40] proposed a method where height and reflection values obtained from terrestrial LiDAR were used to detect soil, crops and weeds. It was reported that the correlation between actual plant heights and LiDAR measured height was R^2^ = 0.75. A strategy was employed in the study to separate areas with vegetation from soil and perform discrimination of monocots, dicots, crops and soil cases [40]. It was found that vegetation presence corresponded to higher reflection values. Therefore, along with LiDAR height measurements, reflection data were used to differentiate soil and vegetation. The predicted values from binary logistic regression were 95.3% for vegetation and 82.2% for soil and there was an overall accuracy of 92.7%. Using canonical discriminant analysis (CDA), the overall success in discriminating these four cases was 72.2%. Soil and dicots were classified with 92.4% and 64.5% accuracy, respectively. The accuracy of the weed discrimination for monocots was poor as they were similar to maize. However, the predictions for the crop were obtained with 74.3% accuracy. The resolution of the images was one of the important factors in this discrimination study, and it is lower if the laser beam footprint size is larger. As a result, the field of view declines with higher accuracy, thereby limiting the efficiency of the proposed method [40]. Hence, selecting the laser beam footprint’s optimal size to discriminate weeds and maze accurately was a challenge in the study.

However, Reji Jayakumari et al. applied a deep convolutional neural network (CNN) model named CropPointNet for the semantic segmentation of crops from a 3D perspective. This model was used on LiDAR point cloud data for object-based classification of cabbage, tomato and eggplant. The crop classification was validated and compared with PointNet and a dynamic graph-based convolutional neural network (DGCNN). It was reported that the crop objects in the 3D point cloud were classified with an overall accuracy of 81.5% using the CropPointNet model. The PointNet and DGCNN had overall accuracies of 55% and 66.5%, respectively. However, CropPointNet, DGCNN and PointNet models discriminated cabbage with 91%, 82% and 72% accuracy, respectively, and eggplant with 88%, 83% and 69% accuracy, respectively. In contrast, the accuracy of discrimination of tomato crop was 65%, 61% and 60% for CropPointNet, DGCNN and PointNet, respectively. It was reported that most of the tomato crop discriminations were underestimated due to apparent confusion with residual soil ridges [41].

#### 3.5.2. Crop Growth Estimation

On the other hand, to measure wheat height, a ground-based multi-sensor system containing LiDAR and ultrasonic sensors was developed by Wenan Yuan et al. [41]. The study also determined the effectiveness of LiDAR, ultrasonic sensors and unmanned aircraft system (UAS) data and compared the results with manual measurement [41]. LiDAR data were pre-processed to correct the slanting effect to improve the accuracy of wheat height estimations. LiDAR had an RMSE of 0.05 m and the correlation between manual and LiDAR had an R^2^ of 0.97; UAS had an RMSE of 0.09 m and the correlation between manual and UAS had an R^2^ of 0.91 for determining canopy height. The RMSE obtained from ultrasonic sensors for height estimation was 0.3 m and the correlation between manual and ultrasonic sensors had an R^2^ of 0.05. It was reported that when the ground is fully covered with vegetation, pre-processing of the point cloud might not be possible in the case of LiDAR due to the possibility of not capturing enough ground point data [42].

Furthermore, to study the effect of nitrogen application in sugarcane growth, Jeremy Sofonia et al. employed a hover map LiDAR utilising simultaneous localisation and mapping (SLAM). The results obtained from LiDAR and photogrammetry were also compared to determine the effectiveness of these two systems [43]. A multispectral camera (for photogrammetry) and LiDAR system mounted to a single unmanned aerial vehicle (UAV) platform were used to obtain the data set regarding the height of sugarcane. The ground returns were detected across the study area throughout all six surveys. The first survey started 58 days after cropping. The interval between surveys was 42 days.

**Figure 5:**
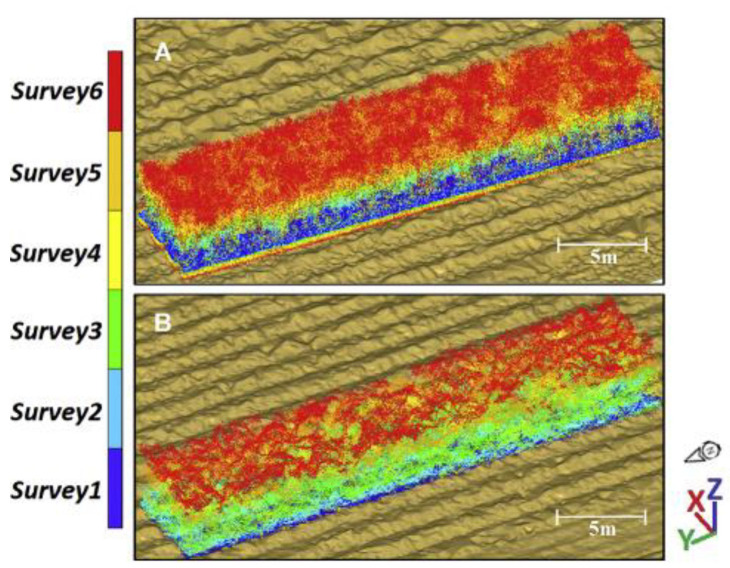
Example (**A**) LiDAR and (**B**) photogrammetry time-series point cloud data coloured by survey (dark blue to red). Reprint from Ref. [43].

On the other hand, the number of ground returns decreased over each survey and was absent from the third survey with photogrammetry. However, using a power regression, the ratio of ground to non-ground returns obtained for LiDAR and photogrammetry had a R^2^ of 0.971 and R^2^ of 0.993, respectively. Figure 5 shows that LiDAR was able to collect more data from Survey6 than photogrammetry. The SLR’s coefficient of determination between LiDAR measured maximum height and photogrammetry-measured maximum height was an R^2^ of 0.885. The coefficient of determination between mean crop height obtained from LiDAR and those obtained from photogrammetry was an R^2^ of 0.929. It was also reported that though both LiDAR and photogrammetry could detect the difference between zero nitrogen and applied nitrogen treatments in the second and third surveys, the ability to detect sugarcane height difference decreased with increasing days after cropping.

#### 3.5.3. Aboveground Fresh Weight Estimation

**Figure 6:**
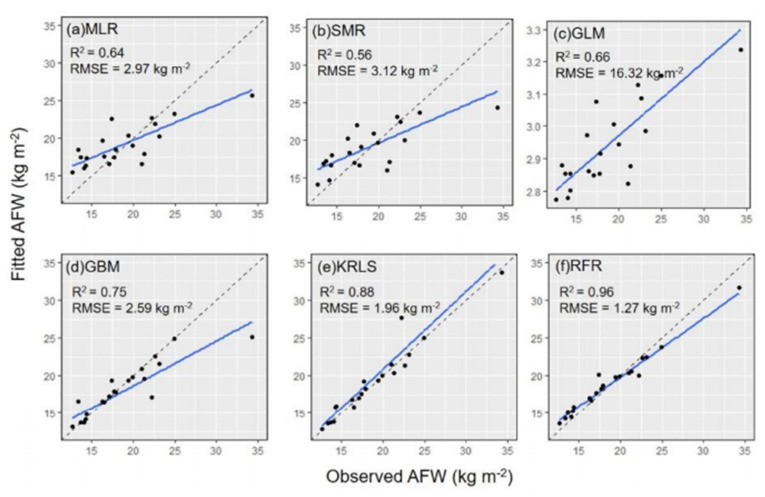
Relationship between observed aboveground fresh weight (AFW) and fitted AFW of sugarcane via different analytical methods. Reprint form Ref. [44].

In addition to measuring sugarcane height, sugarcane aboveground fresh weight (AFW) was also estimated from the correlation between height and weight by Jing-Xian Xu et al. [44], where a LiDAR-based system was mounted on a UAV. In the study, AFW was measured manually; sugarcane height was obtained manually and via LiDAR data. After generating the DEM and DSM from the LiDAR data set, the height of sugarcane was calculated by deducting DSM and DEM raster data. The value of the coefficient of determination between sugarcane height and weight measured in the field on individual plants was 0.51. The weight of sugarcane was positively correlated with its height. Multiple linear regression (MLR), SMR, a generalised linear model (GLM), a generalised boosted model (GBM), kernel-based regularised least squares (KRLS) and RFR algorithms were used to develop the AWF models. A LiDAR data-based machine learning approach was also applied. An evaluation index based on R^2^ and RMSE between fitted and observed data was employed for model accuracy. In machine learning, KRLS, GBM, RFR and GLM were used as predictive models. It was found that RMSE decreased gradually and R^2^ increased gradually in the machine learning methods. The results show that the relationship between observed AFW and fitted AFW via RFR was obtained with an R^2^ of 0.96 and an RMSE of 1.27 kg m^−^^2^, which was the highest value for R^2^ and the lowest value for RMSE among the six models, as shown in Figure 6 [44]. Here, the black dotted line represents the 1:1 line and the solid blue lines represent a linear fit. Table 5 compares various aspects of LiDAR data in weed, crop and soil detection and crop growth estimation.

### 3.6. Spray Drift Measurement

The use of a LiDAR system to characterise drift during pesticide application was also reported in the literature.

#### 3.6.1. Spray Deposition Prediction

Eduard Gregorio et al. demonstrated a LiDAR-based system where lasers of 1.5 μm wavelength were used to design an eye-safe system to monitor pesticide clouds [45]. Parameters including wavelength, emission frequency, pulse energy and reception area were calculated using signal-to-noise ratio simulations for pesticide spray drift measurement. It was reported that the developed LiDAR system could measure mid-range spray drift with distance 2.4 m and temporal (100 ms at maximum pulse repetition frequency) resolution [45].

On the other hand, Emilio Gil et al. used a LiDAR measurement based model to predict spray deposit [46]. The predicted spray deposit obtained from LiDAR data was compared with the deposits measured over the ad hoc test bench [46]. A conventional mist blower with conventional and air injection nozzles and a multi-row sprayer was employed at two airflow rates. The results show that the largest drift fraction was detected within the closest 5 m of the measurement zone for all cases, close to the spray pass and canopy. However, after 5.0 m, a constant reduction in deposition was observed and the reduction was more prominent in the LiDAR measurements. In their experiment, a spray drift cloud exceeding the canopy was scanned using a LiDAR-based system. When the laser beam intercepted the drifting cloud, the sensor detected the angular position and the radial distance of all impacts from the reflected signal. The results show that for a 34,959 m^3^.h^−^^1^ air flow rate with conventional and air injection nozzle types, the correlation coefficient ® between the number of LiDAR detected points and the deposition in the artificial collector placed in the test bench ranged from 0.87 to 0.91 and 0.88 to 0.40, respectively, with a number of repetitions. The correlation coefficient (r) ranged from 0.85 to 0.94 and 0.07 to 0.88 for 27,507 m^3^.h^−^^1^ air flow rate with conventional and air injection nozzle types, respectively, after several repetitions. Furthermore, for a 6423 m^3^.h^−^^1^ air flow rate with conventional nozzle types, the correlation coefficient (r) ranged from 0.93 to 0.98 with a number of repetitions. In this study, it was reported that the droplet size could have an influence on drift measurements using LiDAR.

#### 3.6.2. Different Aerosol Detection

Moreover, Eduard Gregorio et al. used a polarisation LiDAR to detect different agricultural aerosol emissions [47]. In their study, a 355 nm polarisation Lidar system was used to determine the emissions generated during pesticide spraying activities. The depolarisation ratios caused by road dust, field dust, diesel exhaust and pesticide spray drift, were 0.385, 0.220–0.268, 0.099 and 0.028–0.043, respectively. Table 6 presents the comparison of various aspects of LiDAR data in spray drift measurement.

### 3.7. Soil Property Detection

A LiDAR system can also be used to predict soil properties such as moisture and roughness, as reported in the literature.

#### 3.7.1. Soil Moisture Prediction

The applications of LiDAR data to detect soil property were also reported. Florence Margaret Southee et al. studied three LiDAR-derived terrain surfaces generated at different spatial resolutions to determine the resolution that best characterises measured soil moisture patterns [48]. In their study, topographic wetness index (TWI), per cent elevation index (PEI) and canopy height model (CHM)) at spatial resolutions 2 m, 5 m, 10 m and 20 m were applied to determine the resolution that best characterised the soil moisture [48]. Depression removal algorithms were also used. The coefficients of determination between soil moisture and TWI for 0–15 cm and 0- 40 cm depths were an R^2^ of 0.346 and an R^2^ of 0.292, respectively. The presented results suggest that high-spatial-resolution variables (2 m and 5 m) might be more effective in modelling soil moisture trends at shallow depths (0 to 15 cm). On the other hand, coarser resolutions (10 m, 20 m) might be more suitable at greater depths (0 to 40 cm).

**Figure 7:**
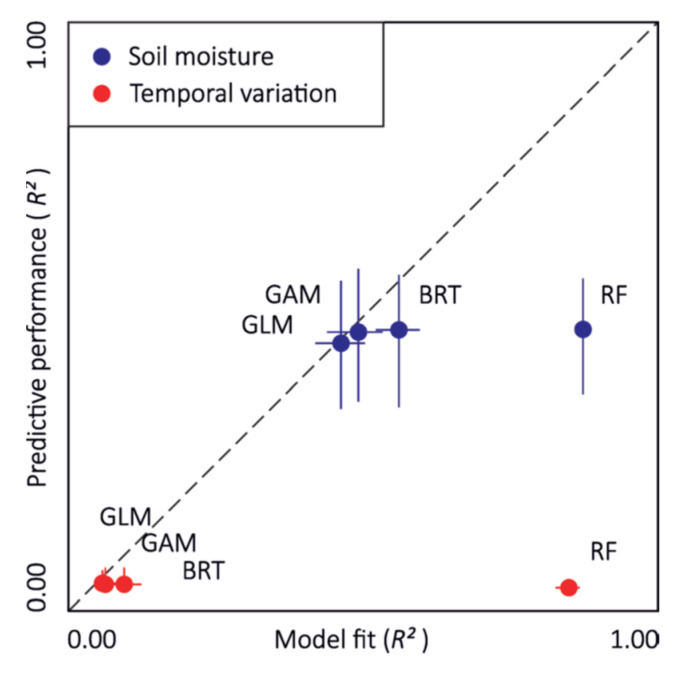
Comparing four soil moisture modelling methods. Reprinted with permission from Ref. [49] Copyright 2017, Wiley.

On the other hand, Julia Kemppinen et al. studied the significance of soil and land surface properties in landscape-scale soil moisture variation using LiDAR data and field investigations [49]. In their study, GLM, generalised additive models (GAM), boosted regression trees (BRT) and RFR were applied to model soil moisture and its temporal variation. Figure 7 presents a plot of the predictive performances of four soil moisture modelling methods. The horizontal and vertical segments represent the ranges of each modelling method. Results indicate that the average model fit had R^2^ = 0.60 and RMSE 8.04 VWC% and predictive performances were R^2^ = 0.47 and RMSE 9.34 VWC%. The temporal variation models demonstrated a fit of R^2^ = 0.25 and RMSE 13.11 CV% and predictive performances were R^2^ = 0.01 and RMSE 15.29 CV%. Thus, it was concluded that in high-latitude landscapes, soil and land surface properties deserve importance.

#### 3.7.2. Soil Roughness Prediction

In addition to modelling soil moisture, soil surface roughness could also be determined from LiDAR data. Russell Turner et al. used an airborne LiDAR [50] to determine the surface roughness (SR) of agricultural soil and evaluate the LiDAR data’s accuracy. In their study, surface heights, root mean square (RMS) and correlation length (CL) over soils with variable tillage conditions were considered. The results demonstrate the correlation between LiDAR-estimated and ground-measured RMS estimated with R^2^ > 0.68 and up to 0.88. Table 7 presents the comparison of different aspects of LiDAR data in soil property detection.

### 3.8. Yield Prediction

The application of LiDAR data for yield estimation was also reported. James P. Underwood et al. used a mobile terrestrial LiDAR scanner (MTLS) for almond orchards [51]. Canopy volume was calculated from the 3D models and a classification procedure was used to estimate flower and fruit density. These schemes were compared to each tree harvest weight in estimating yield. LiDAR canopy volume had a relationship for 39 tree samples to yield with R^2^ = 0.77. At the same time, hand-held photography and image analysis were employed and fruit density presented an R^2^ of 0.71 [51].

**Figure 8:**
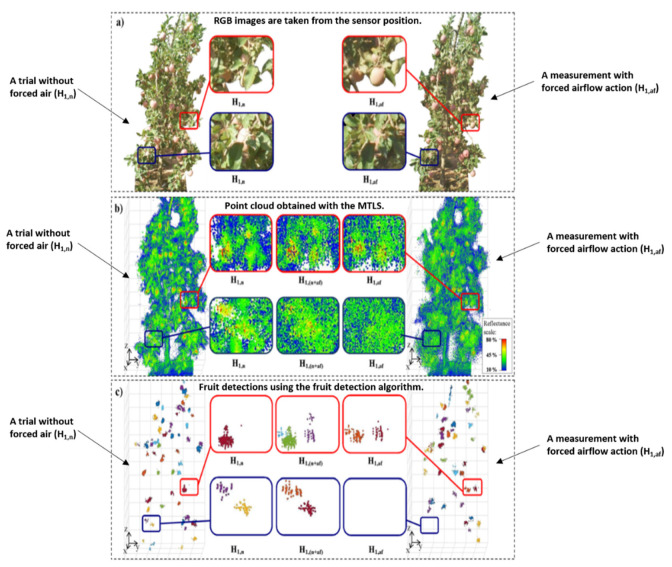
Illustration of the forced airflow effect in fruit detection. Reprinted with permission from Ref. [52] Copyright 2019 Elsevier.

On the other hand, a multi-beam LiDAR sensor with forced airflow with an air-assisted sprayer was used to detect apple fruit and predict its yield by Jordi Gené-Mola et al. [52]. Reflectance thresholding (RT) and a support vector machine (SVM) were employed to develop an algorithm to detect fruits in their study. The experiment was based on the assumption that using a multi-view sensor and moving the tree foliage would increase fruit detection and reduce fruit occlusions. Figure 8 presents the effect of the forced airflow in fruit detection. The analysis between the number of apples detected with LiDAR with the forced airflow system and the actual number of apples per tree had RMSEs of 19.0% and 12.4 % and R^2^ of 0.58 and 0.54 when scanning was performed from the east and west sides, respectively. However, when both tree sides were considered, the obtained R^2^ was 0.87 with an RMSE of 5.7% [52]. Table 8 presents the comparison of various aspects of LiDAR data in yield prediction.

### 3.9. Crop Damage Detection

It was also reported that LiDAR could be used to detect crop damage. Longfei Zhou et al. used LiDAR data of lodged maize using an UAV to analyse the relationship of the plant height and degrees of lodging [53]. The effect of different degrees of lodging on maize plant ability to recover height was also presented in their study. The CHM, DEM and DSM were used in the analysis. The maize plant height estimated using LiDAR and the measured plant height obtained using a telescopic levelling ruler were used to verify the plant height estimated using UAV-LiDAR. The inaccuracy of the obtained plant height from LiDAR data widened with increased actual plant height. The results had R^2^ of 0.964, RMSE of 0.127 and a normalised root mean square error (nRMSE) of 7.449%. The study also found that the plant height recovered to various degrees in all lodging areas. Table 9 below presents the various LiDAR estimations regarding the canopy heights. It was reported that the textures varied and the plant heights differed because of the inconsistent recovery ability of each maize lodging type [53].

## 4. Data Processing

No review of research papers on LiDAR would be complete without referring to the pre-processing avenues of the substantially noisy LiDAR data, which are critical to removing unwanted information and finding the recommended value of the data’s spatial resolution (points/m^2^).

### 4.1. Pre-Processing of LiDAR Data

Qi et al. published [54] a pre-processing strategy on 3D LiDAR data based on outlier removal through distance normalisation to offset the effect of spatial resolution changing with distance and wake filtering. The point cloud was shrunk through vertical attributes of wave wake and obstacle surface, and wake plane estimation and wake point removal were completed via the random sample consensus (RANSAC) method [55]. It was demonstrated through experimentation that their algorithm maintained the required values while effectively removing non-obstacle points, although with an increased average time consumption of 23.3 ms. Their recall rate was 95.4%, and the F1 score, which is the balance of non-obstacle points filtering and obstacle points preservation, was 93.7%.

Sibel Canaz Sevgen employed pre-processing on LiDAR data to clean noise and duplicate values before generating 12 features and a subsequent random forest (RF) classification [56]. Ground truth data were subsequently obtained from aerial photographs of a complex urban area to classify buildings, trees, asphalt roads and the ground with respective accuracies of 77.90%, 58.37%, 72.90% and 71.53%. A ground point extraction automated algorithm based on the height difference of ground points and non-ground points for each point on three LiDAR data sets was proposed and evaluated quantitatively and qualitatively by Sevgen et al. [57]. The overall accuracies were calculated to be 95%, 97%, and 98% for the three LiDAR data sets. The datasets had different point densities, vegetation densities and building data values. Ozdemir et al. [58] utilised a data-preprocessing technique to extract trees from the LiDAR point cloud including the density-based spatial clustering of applications with noise (DBSCAN) algorithm [59] and cloth simulation filtering (CSF) method [60] for clustering and filtering, respectively. DBSCAN was also employed in [61] along with the mean shift algorithm for the automated detection of single street trees, where the ground truth data were derived through field investigations. They achieved high completeness and correctness values for two test areas and clustering methods. There are other data filtering methods available that are slope-based [62], mathematical-morphology-based [63], surface-based [64], etc. The performances of widely employed filtering algorithms were compared in [65,66,67].

### 4.2. Recommended Value of the Spatial Resolution (Points/m^2^)

Sanchez-Diaz et al. developed [68] a canopy height model for shade trees in cocoa agrosystems from LiDAR data with a resolution of 0.47 points/m^2^ and measured values in the field. It was validated using the coefficient of determination (R^2^), mean absolute error (MAE) and root mean square error (RMSE). However, [68,69,70] recommend that a LiDAR point cloud with a minimum spatial resolution of ≥0.50 points/m^2^ be utilised to extract better information through analysis. Better results regarding other vegetation attributes, e.g., canopy diameter and tree species identification in addition to canopy height, could have been produced by [68] had they used LiDAR data with a spatial resolution of ≥0.50 points/m^2^.

## 5. Future Research Directions

This review presents different applications of LiDAR data. From a study of the literature, there is also room for much more research in this vast field of LiDAR. It is important to develop a LiDAR-based system that can present information closest to the actual values. There is also demand for developing software and models to analyse LiDAR data accurately [11], efficiently and economically.

One of the crucial factors is the resolution of images obtained from LiDAR data [40]. The angular resolution, laser beam footprint and scanning window play an essential role in resolving resolution. High denser points and smaller laser beam width acquire greater angular resolution. Furthermore, if the laser beam footprint’s size is smaller, the resolution of images will be higher. When the scan window increases, the beam width enlarges and the point density decreases. This results in the actual angular resolution being lowered [31,71]. Therefore, research can determine the optimum angular resolution, laser beam footprint, scan window and other methods to obtain images from LiDAR data with high resolution. Thus, accurate and precise information regarding agricultural landscaping can be obtained from high-resolution images.

It was reported that LiDAR point density affected vegetation biophysical parameter estimation accuracy, which was not highly accurate [72]. Therefore, researchers could also start addressing the optimum LiDAR point density to predict optimum estimation results. It is also essential to increase the horizontal resolution of LiDAR scanning to avoid overestimation, for example, in determining LAI [33].

It was also found that acquiring accurate topographic information was a challenging task [28]. Furthermore, for high-vegetation sites, there is a possibility that LiDAR cannot capture enough ground point data to calculate height, which is a feature to distinguish between weeds and crops [42]. Therefore, there is also room for research to develop methods where accurate information can be obtained in high-vegetation sites.

Plant geometry is also vital in obtaining plant phenotype and biomass information [35,39] from LiDAR data. However, complex plant geometry can cause errors and affect the accuracy of LiDAR-based systems. Therefore, research can be focused on developing methods where phenotype and biomass information of plants with complex geometry can be acquired accurately. Moreover, the scan window is also a critical factor in achieving accurate information, for example, regarding landscaping [10]. The LiDAR scan window size would vary with plant geometry, spacing and the DSM’s resolution. Hence, determining the optimum scan window to study plants and landscaping could lead to another research topic.

Future research could also focus on developing a LiDAR-based system where the system is less costly, data acquisition is less time-consuming, the data analysis method provides accurate information and the overall system is user-friendly. SiLC is designing chips with frequency-modulated continuous wave (FMCW) LiDAR capable of velocity detection [73]. Sony is innovating a USD 120 single chip for LiDar that houses single-photon avalanche diode (SPAD) depth sensors [74]. Stanford University is planning to build a commercial LiDAR system based on the piezoelectric effect that can capture megapixel-resolution 3D depth maps using standard digital cameras [75].

## 6. Conclusions

This manuscript extensively reviews a number of LiDAR-based techniques in agricultural applications. High-quality research papers and articles published during the last one and a half decades (i.e., from 2008 to 2022) from academia and industry were evaluated and reported. Before starting the discussion about real-life implementations, an overview of LiDAR technology was presented. A total of nine application areas and their sub-areas were presented: landscape and topography; leaf area index and canopy volume; crop biomass estimation; canopy phenological stages and phenotype characterisation; weed, crop and soil detection and crop growth estimation; spray drift measurement; soil property detection; yield prediction; and crop damage detection. Nine tables, one at the end of each section, captured the summaries of significant aspects of the papers. These tables presented the key features of the papers, e.g., LiDAR type, algorithms, models, classification, analytical methods, etc. Afterwards, data processing was discussed, as this is an essential aspect of noisy and substantial-sized LiDAR data. Future research directions pertaining to LIDAR data in the agricultural context were presented at the end.

## Figures and Tables

**Table 1:** Comparison of various aspects of LiDAR data in determining agricultural landscape and topography.

LiDAR Type	Algorithm	Model	Classification Method	Features	Results
Airborne	DWT; watershed algorithm		Classification and regression trees	Elevation profiles; concavity indicators	For DWT and watershed, overall accuracy: ~71%. Mean ditch omission rate: ~50%. Mean ditch commission rate: ~15 % [28].
Airborne	SCM	DEM; GIS	Quantile classification	Top of canopy height; ground height; flat to low slope area	LiDAR data based SCM estimated less than the GIS model [29].
Airborne		DEM		Slope; flow accumulation; stream power index	3 m LiDAR identified a higher number of most prominent gullies compared to 30 m LiDAR [30].
Airborne	Fusion’s ground filter algorithm	DTM; DSM; nDSM	Focal statistics function; iterative self-organising data analysis technique	Vine rows height; relative elevation of surface features from ground level	Mean accuracy of correctly classified vineyard area: 97.55%. Mean accuracy of parcel delineation: 88.79% [31].
Airborne Terrestrial		DTM		Relative path impact index (RPII)	The RPII obtained from 0.2 m TLS DTM presented more accurate results compared to RPII obtained from 1 m ALS DTM [10].
Airborne		DEM; SSR; GRASS; GIS r.sun; SLR		Linke turbidity index; Julian day; time-step; LPI	R^2^ between SSR and field measurement for total solar radiation: 0.92. R^2^ between GLA and field measurement for total solar radiation: 0.692. R^2^ between LPI and canopy openness obtained from GLA: 0.768 [32].

**Table 2:** Comparison of various aspects of LiDAR data in measuring leaf area index and canopy volume.

LiDAR Type	Analytical Method	Classification Algorithm	Features	Results
Terrestrial	SLR		TAI; LAI	R^2^ between plant volume and LAI: 0.8422 (pear tree), 0.814 (apple tree) and 0.8058 (vineyards). R^2^ between TAI and LAI: 0.9194 (vineyards). TAI could be used as a parameter to determine LAI for some specific crops in a vineyard [19].
Terrestrial	Lillefors tests; Box–Cox test; Pearson’s product- moment correlation coefficient		Canopy height; crop width; canopy volume; LAI	R^2^ between LAI and canopy volume using ultrasonic: 0.51; between LAI and canopy volume using LiDAR: 0.21; R^2^ of canopy volume obtained from LiDAR and ultrasonic sensors: 0.56 [11].
Terrestrial	Vector map; raster map	Fuzzy c-means	LAI	The LiDAR system could be used intermittently if the maximum distance between scans along the rows did not exceed 15 m with a scan length of 1 m [33].
Terrestrial	Poisson distribution; SLR		LAI; TAI; tree height; cross- sectional area; canopy volume	R^2^ between TAI and LAI: 0.92; between canopy volume and LAI: 0.81; between cross-sectional area and LAI: 0.72; between tree height and LAI: 0.62 [12].
Terrestrial	SLR		MSA; LAI	R^2^ between MSA and LAI: 0.798 [34].

**Table 3:** Comparison of various aspects of LiDAR data in crop biomass estimation.

LiDAR Type	Analytical Method	Model	Features	Results
Terrestrial	SLR; PMCC	3D volumetric model	The volume of woody tissue of vines	Volumes calculated from LiDAR data range between 1.31 and 10.61 L. Volumes obtained from analogue measurement range between 0.83 and 5.05 L. The SLR of analogue volume on LiDAR-based volume indicates that slope values range from 0.43 to 0.54. Furthermore, the PMCC ranges between 0.73 and 0.97 [35].
Terrestrial	SLR; LSR; SMR; ANN; RFR		Height; canopy cover; canopy volume	At the plot level: LSR and SLR had R^2^ of 0.79 and 0.80, respectively. The R^2^ of SMR, ANN and RF were 0.80, 0.68 and 0.79, respectively. At the individual plant level: LSR and SLR had R^2^ of 0.93 and 0.96, respectively. The R^2^ of SMR, ANN and RF were 0.94, 0.93 and 0.94, respectively. In the leaf group level: LSR and SLR had R^2^ of 0.95 and 0.92, respectively. The R^2^ of SMR, ANN and RF were 0.97, 0.97 and 0.97, respectively. At the stem level: LSR and SLR had R^2^ of 0.93 and 0.94, respectively. The R^2^ and RMSE of SMR, ANN and RF were 0.94, 0.95 and 0.95, respectively [14].
Terrestrial	SLR	DSM; DTM	Vegetation volume; NNI; canopy height; canopy volume	Relationships between observed physical proxies and LiDAR-derived vegetation volume for all seasons and growth stages were with R^2^ >= 0.72. The range of relationships between the actual nitrogen concentration and green laser return intensity was R^2^ = 0.10–0.75 [36].
Terrestrial	Percentile algorithm; Pearson’s correlation coefficient (r); SLR		AGB; CH; LPV; LCH	The correlations between AGB and LPV were up to r = 0.86. The correlations between CH and LCH were up to r = 0.94 [37].

**Table 4:** Comparison of various aspects of LiDAR data in canopy phenological stages and phenotype characterisation.

LiDAR Type	Analytical Method	Model	Features	Results
Terrestrial	SLR	GRASS-GIS	Canopy height; canopy width; LAI; TRV; LWA	R^2^ between manual and LiDAR scan of canopy height, between manual and LiDAR scan of canopy width, between TRV and the growth stage and between LWA and the growth stage were 0.98, 0.81, 0.99 and 0.95, respectively [16].
Terrestrial	SLR; distance-based clustering	DTM	Plant height; PAI; PLA; plant area density (PAD)	R^2^ between plant height and manual measurement, between PLA and manual measurement and between PAI and manual measurement were 0.96, 0.92 and 0.70, respectively [17].
Terrestrial	SLR; ANOVA test analysis		Plant volume; canopy height; PCA	R^2^ between manual and LiDAR measurements of canopy height, between manual and LiDAR measurements of PCA, between manual and LiDAR measurements of canopy volume were 0.97, 0.97 and 0.98, respectively [38].
Terrestrial	SLR	Delaunay triangulation algorithm; K-means clustering; LOWESS	Leaf area; plant area; the inclination angle of individual leaves; leaf angular distribution of the whole plant	R^2^ between model-derived leaf area and the reference measurement for maize was 0.92. For sorghum, R^2^ between model-derived leaf area and the reference measurement was 0.94. For maize, R^2^ between leaf inclination angles measured from 2D images and those obtained from the 3D model was 0.904. For sorghum, R^2^ between leaf inclination angles measured from 2D images and those obtained from the 3D model was 0.723 [37].

**Table 5:** Comparison of LiDAR data in weed, crop and soil detection and crop growth estimation.

LiDAR Type	Analytical Method	Model	Features	Results
Terrestrial	SLR; binary logistic regression; CDA		Plant height; reflection value	R^2^ between LiDAR measured height and actual plant heights was 0.75. The predicted values from binary logistic regression shows an accuracy of 95.3% for vegetation and 82.2% for non-vegetation/soil, with an overall accuracy of 92.7%. Using canonical discriminant analysis (CDA), the overall success to discriminate was 72.2%. The soil and dicots were classified with 92.4% and 64.5% accuracy, respectively [40].
Terrestrial		CropPointNet; PointNet; DGCNN	Crop height	CropPointNet model had an overall accuracy of 81.5%. PointNet and DGCNN had overall accuracies of 55% and 66.5%, respectively. CropPointNet, DGCNN and PointNet models discriminated cabbage with 91%, 82% and 72% accuracy, respectively, eggplant with 88%, 83% and 69% accuracy, respectively, and tomato crop with 65%, 61% and 60% accuracy, respectively [41].
Terrestrial	SLR		Canopy height	R^2^ between LiDAR measured height and manual measurement, between UAS measured height and manual measurement and between ultrasonic-sensor-measured height and manual measurement were 0.97, 0.91 and 0.05, respectively [42].
Airborne	Power regression; SLR	DTM	Sugarcane height	The ratio of ground to non-ground returns with LiDAR had R^2^ = 0.971. The ratio of ground to non-ground returns with photogrammetry had R^2^ = 0.993. R^2^ between maximum crop height obtained from LiDAR and those obtained from photogrammetry was 0.885. R^2^ between the mean crop height obtained from LiDAR and those obtained from photogrammetry was 0.929 [43].
Airborne	MLR; SMR; GLM; GBM; KRLS; RFR	DEM; DSM	AFW	R^2^ between observed AFW and fitted AFW via RFR was 0.96, the highest value for R^2^ among the six models [44].

**Table 6:** Comparison of various aspects of LiDAR data in spray drift measurement.

LiDAR Type	Analytical Method	Features	Results
Terrestrial	Signal to noise ratio simulations	LiDAR signal backscatters signal to noise ratio	LiDAR system measured mid-range spray drift with distance 2.4 m and temporal (100 ms at maximum pulse repetition frequency) resolution [45].
Terrestrial	Linear function	Number of drift drops	For 34,959 m^3^.h^−1^ air flow rate: The correlation coefficient ranged from 0.87 to 0.91 with conventional nozzle types. The correlation coefficient ranged from 0.88 to 0.40 with air injection nozzle types. For 27,507 m^3^.h^−1^ air flow rate: The correlation coefficient ranged from 0.85 to 0.94 with conventional nozzle types. The correlation coefficient ranged from 0.07 to 0.88 with air injection nozzle types. For 6423 m^3^.h^−1^ air flow rate: The correlation coefficient ranged from 0.93 to 0.98 with conventional nozzle types [46].
Terrestrial (polarisation)	Polarisation LiDAR methodology	Volume depolarisation ratio; particle depolarisation ratio	The results show that particle depolarisation ratios due to field dust (0.220–0.268) and road dust (0.385) were higher than those caused by pesticide spray drift (0.028–0.043) or diesel exhaust (0.099) [47].

**Table 7:** Comparison of various aspects of LiDAR data in soil property detection.

LiDAR Type	Analytical Method	Model/ Algorithm	Features	Results
Airborne	Shapiro–Wilk test; Brown– Forsythe test; repeated measures analysis of variance; SLR	Depression removal algorithms; DSM; DEM; impact reduction approach algorithm	TWI; PEI CHM	R^2^ between soil moisture and TWI for 0–15 cm depth was 0.346. R^2^ between soil moisture and TWI for 0–40 cm depth was 0.292. High-spatial-resolution variables (2 m and 5 m) might be more effective in modelling soil moisture trends at shallow depths (0 to 15 cm). Coarser resolutions (10 m and 20 m) might be more suitable at greater depths (0 to 40 cm) [48].
Airborne	SLR		Surface heights; root mean square (RMS)	Correlation between LiDAR estimated and ground-measured (RMS) estimated had R^2^ > 0.68, up to 0.88 [50].
Airborne	GLM; GAM; BRT; RFR	Soil moisture model; temporal variation model; DTM	TWI; system for automated geoscientific analyses; soil wetness index; topographic position index	The average model fit of the soil moisture model had R^2^ = 0.60. The temporal variation model had a fit of R^2^ = 0.25 [49].

**Table 8:** Comparison of various aspects of LiDAR data in yield prediction.

LiDAR Type	Analytical Method	Model/ Algorithm	Features	Results
Terrestrial	SLR	3D point cloud segmentation and voxelization	Canopy volume; flower density; fruit density	LiDAR canopy volume had a relationship to yield with an R^2^ of 0.77. Hand-held photography and image processing to measure fruit density presented an R^2^ of 0.71 [51].
Terrestrial	SLR	SVM; RF; density-based scan algorithm	Mean canopy height; canopy width; contour cross-section area	LiDAR with forced airflow system and the actual number of apples per tree had RMSEs of 19.0% and 12.4 % and R^2^ of 0.58 and 0.54 when scanning from east and west sides, respectively. LiDAR with forced airflow system and the actual number of apples per tree presented R^2^ of 0.87 when using data from both tree sides [52].

**Table 9:** The range of canopy height estimated using LiDAR.

No Lodging	With Lodging		
	Stem Tilt	Stem Folding	Root Lodging
2.01~2.28 m	1.21~1.47 m	0.06~0.17 m	0.08~0.18 m

## Data Availability

No new data were created or analysed in this study. Data sharing is not applicable to this article.
